# Hepatic DNA Damage Induced by Electronic Cigarette Exposure Is Associated With the Modulation of NAD+/PARP1/SIRT1 Axis

**DOI:** 10.3389/fendo.2019.00320

**Published:** 2019-06-04

**Authors:** Jorge Espinoza-Derout, Xuesi M. Shao, Emmanuel Bankole, Kamrul M. Hasan, Norma Mtume, Yanjun Liu, Amiya P. Sinha-Hikim, Theodore C. Friedman

**Affiliations:** ^1^Division of Endocrinology, Metabolism and Molecular Medicine, Department of Internal Medicine, Charles R. Drew University of Medicine and Science, Los Angeles, CA, United States; ^2^David Geffen School of Medicine, University of California, Los Angeles, Los Angeles, CA, United States

**Keywords:** reactive oxygen species, DNA damage, hepatic metabolism, mitochondria, NAD+

## Abstract

The prevalence of electronic cigarette (e-cigarettes) use has rapidly increased worldwide. Use of tobacco products has been associated with DNA damage and metabolic syndrome. Using Apolipoprotein E knockout (ApoE^−/−^) mice on a western diet (WD), a mouse model of non-alcoholic fatty liver disease (NAFLD), we recently demonstrated that nicotine in e-cigarettes activates hepatocyte apoptosis, and causes hepatic steatosis. This study examines the harmful effects of e-cigarettes on the liver with a special emphasis on DNA damage and mitochondrial dysfunction. ApoE^−/−^ mice were exposed to saline, e-cigarettes without nicotine or e-cigarettes with 2.4% nicotine for 12 weeks using our newly developed mouse e-cigarette exposure model system that delivers nicotine to mice leading to equivalent serum cotinine levels found in human cigarette users. Mice exposed to e-cigarette (2.4% nicotine) had increased apurinic/apyrimidinic (AP) sites, a manifestation of DNA damage. Additionally, e-cigarette (2.4% nicotine) produced a decrease in NAD+/NADH ratio and increased oxidative stress in hepatic cells, in comparison with saline and e-cigarette (0%). Western blot analysis showed that mice treated with e-cigarette (2.4% nicotine) had increased poly (ADP ribose) polymerase (PARP1) activity associated with reduced levels of Sirtuin 1 (SIRT1). Furthermore, mitochondrial DNA mutations and PTEN-induced kinase 1 (PINK1) were increased in mice treated with e-cigarette (2.4% nicotine). Transmission electron microscopy revealed that hepatocytes of mice treated with e-cigarette (2.4% nicotine) exhibited increased vacuolization of the mitochondria and a reduction in cellular organelles. These results demonstrate the adverse effects of e-cigarettes exposure leading to NAD+ deficiency which may suggest a mechanistic link between e-cigarette-induced hepatic DNA damage and mitochondrial dysfunction.

## Introduction

In 2004, a Beijing-based company, Ruyan Group (Holdings) Ltd, China, patented and launched electronic cigarettes (e-cigarettes) also called electronic nicotine delivery systems (ENDS) (1), that delivers nicotine to users without burning tobacco (2). E-cigarettes are battery-powered devices resembling a cigarette that contains a microelectrical circuit activated by drawing on the mouthpiece (3). People report buying them to help quit smoking, to reduce cigarette consumption and costs, to relieve tobacco withdrawal symptoms due to workplace smoking restrictions, or as a replacement for cigarette smoking (4, 5).

DNA damage generated by oxidative stress has a strong mechanistic link to the pathophysiology of metabolic disease (6). Acute side-stream tobacco produces oxidative DNA damage in the liver of mice (7) and e-cigarettes have been reported to induce DNA damage in lungs, heart, and bladder of mice (8). As part of the DNA repair machinery, Poly (ADP-ribose) polymerases (PARP) family of proteins is activated by DNA damage. PARP1 account for approximately 90% of this family protein activity (9). Nicotinamide adenine dinucleotide (NAD+) is consumed by PARP1 to transfer the ADP-ribose moiety onto target proteins.

NAD+ homeostasis is regulated by nutrient-sensing signaling pathways, which play key roles in metabolism and survival. PARP1 and Sirtuin-1 (SIRT1) have a common co-factor, NAD+. Therefore, PARP1 activity can impact SIRT1 activity by reducing the NAD+ pool (6). Energy shortage increases NAD+ biosynthesis and SIRT1 activity and reduces PARP-1 activity in animals (10). Since PARP1 and SIRT1 have a role in DNA repair and metabolism, they have been proposed as a point of convergence for this process (11).

Increased reactive oxidant species (ROS) contributes to DNA damage and age-related disorders that activate PARP1 activity (12). ROS induced-DNA damage reduces NAD+ levels by increasing PARP1 activity. PARP1 activity is enhanced with aging (13) and high caloric intake (10). Additionally, PARP1 inhibition provides protection against diet-induced obesity (14). NAD+ is increased up to 2-fold in Parp1-null mouse tissues.

Mitochondria are the leading producers of ROS; and ROS can produce mitochondrial damage especially in highly susceptible DNA. Mitochondria DNA mutations and mitochondrial dysfunction have been associated with hepatic steatosis, metabolic syndrome, and obesity (15). Consistently, mouse models and humans with metabolic disease develop DNA damage in both genomic (6) and mitochondrial DNA (mtDNA) (16). PARP1 not only regulate mitochondrial activity by modulating NAD+ pools and SIRT1 activity, but also, PARylation produces depolarization of mitochondrial membrane potential (17), with a consequent PTEN-induced kinase 1 (PINK1) stabilization in damage mitochondria (18).

In previous studies, we have shown that intraperitoneal injections of nicotine when combined with a high-fat diet (HFD) triggers greater oxidative stress, activates hepatocyte apoptosis, and amplifies hepatic steatosis (19, 20). The synergistic effects of HFD and nicotine were linked with the inactivation of AMP-activated protein kinase (AMPK) and stimulation of lipogenesis through the activation of Acetyl-CoA carboxylase (19, 20).

The effects of DNA damage on metabolism is an area of intense research. Using Apolipoprotein E knockout (ApoE^−/−^) mice on a western diet (WD), a mouse model of non-alcoholic fatty liver disease (NAFLD), we recently demonstrated that e-cigarettes containing nicotine trigger great oxidative stress, activate hepatocyte apoptosis, and cause hepatic steatosis (21). In that study, we used our newly developed mouse e-cigarette exposure model system that delivers nicotine to mice leading to equivalent serum cotinine levels found in human cigarette users. We elected to use ApoE^−/−^ mice, as these animals, when fed a WD with 45% fat, as opposed to a very HFD with 60% of calories derived from fat develop hepatic steatosis (22). These mice on a WD represent a novel and fast model with all characteristics feature of non-alcoholic steatohepatitis (NASH) and metabolic syndrome, including abnormal glucose tolerance, hepatomegaly, hepatic steatosis, liver fibrosis and inflammation (23). Furthermore, because NAFLD has been identified as an independent risk factor of atherosclerosis and cardiovascular disease (24, 25), an additional advantage of this model is that it is an ideal model for linking atherosclerosis and NAFLD.

This study examines the harmful effects of e-cigarettes on the liver with a special emphasis on DNA damage and mitochondrial dysfunction. Our results indicate that e-cigarette exposure produces decreased levels of hepatic NAD+ associated increased oxidative stress, PARP1 activation, and mitochondrial dysfunction and may suggest a possible link between DNA damage and metabolic disease including NAFLD induced by e-cigarettes exposure.

## Methods

### Animals

This study was carried out in accordance with the recommendations in the Guide for the Care and Use of Laboratory Animals of the National Institute of Health. The Institutional Animal Care and Use Committee on the Ethics of Animal Experiments of Charles R. Drew University of Medicine and Science approved the protocol. Mice were housed under controlled temperature (22°C) and photoperiod (12-h light and 12-h dark cycle). Male ApoE^−/−^ mice (C57BL/6J background) were purchased from Jackson Laboratory (Bar Harbor, ME) and at 8 weeks of age, mice were started on a WD (D12079B; Research Diets, New Brunswick, NJ) and were exposed to e-cigarette aerosol from bluCig PLUS Classic tobacco e-cigarettes containing 2.4% nicotine (purchased on the bluCig website) for 12 weeks. For controls, mice were exposed to a bluCig PLUS e-cigarette containing Gold Leaf tobacco (0% nicotine) or saline aerosol (27) (Afasci Inc, Burlingame, CA). Classic tobacco and Gold Leaf tobacco flavors are similar except for their nicotine content.

### E-Cigarettes Delivery System

We established an e-cigarette aerosol generation and rodent exposure system (patent number PCT/US17/54133). The system includes an aerosol exposure chamber that holds up to 5 free-moving mice, e-cigarette holders and the e-cigarette activation control unit. The device is connected to a pressurized air source and we adjust the air pressure that activates e-cigarettes and generates an appropriate flow of e-cigarette aerosol to the mouse exposure cage. The number of activated e-cigarettes and the air-flow rate control the e-cigarette aerosol concentration. The air pressure and flow are continuously monitored with pressure gauges and flow meters during experiments. Since e-cigarette activation is intermittent, fresh air-flow is maintained when e-cigarette aerosol flow stops that eliminates residue aerosol from the chamber and provides fresh air for the mice. Our chronic intermittent e-cigarette exposure protocol was: activation for 4 s is a puff; 8 puffs per vaping episode with an inter-puff interval of 20 s; one vaping episode every 30 min. Mice were exposed to intermittent e-cigarette aerosol for 12 h (“on”) 21:00–09:00 the next morning with a 12 h “off” period 09:00–21:00. Batteries were charged every day and were replaced every other week. During the light phase of 12 h. (09:00–21:00), mice were returned to their home cages, no aerosol was delivered and the e-cigarette chambers were cleaned. Food and water were provided *ad lib* during both light and dark phases.

### Oxidative DNA Damage Analysis

Genomic DNA was isolated using the genomic-tip 20/G (Qiagen, #10223) and DNA Buffer Set (Qiagen, #19060). The detection of apurinic/apyrimidinic (AP) sites was performed using an aldehyde-reactive probe (ARP) kit (Kamiya Biomedical, #DN-002), according to the manufacturer's instructions. Data were expressed as the number of AP sites per 10^6^ nucleotides, as determined using a standard provided by the manufacturer.

### Western Blot Analyses

The liver tissue was homogenized using T-PER Tissue Protein buffer (Thermo Fisher, 78510), supplemented with protease inhibitors (Thermo Fisher, #87785) and proteins were separated by polyacrylamide electrophoresis and transferred onto nitrocellulose membrane as described (26). The membranes were blocked in Tris-buffered saline/0.1% Tween 20 with 5% bovine serum albumin powder for 1 h. After incubation with the primary antibody, detection was performed using secondary horseradish peroxide–coupled ECL Western Blotting Substrate (Pierce, #32106). The following antibodies were used: rabbit anti-beta actin (Abcam, #ab8227), rabbit anti–PARP-1 (Cell signaling, #9542), mouse monoclonal anti-PAR (Trevigen, #4335-MC-100), rabbit anti–SIRT1 (Abcam, #ab12193), and rabbit anti–PINK1 (Proteintech, #23274-1-AP). The quantification of the Western blots was performed with the ImageJ program (NIH, Bethesda, MD).

### NAD+/NADH Ratio Determination in Hepatocytes

Assessment of NAD+ and NADH and their ratio was performed using a quantitative colorimetric assay (Bio Assay systems, #E2ND-100), according to manufacturer's instructions. The absorbance of the reaction mixture was measure at 565 nm and normalized by the total protein.

### TBARS Measurement

After washed with PBS, samples were homogenized and sonicated in RIPA buffer supplemented with protease inhibitors (Roche #4693159001). TBARS were measured using a TBARS (TCA Method) Assay Kit (Cayman Chemical, #700870), following the manufacturer's protocol for fluorometric measurements.

### Quantitative PCR Assay for Mitochondrial DNA Damage

The genomic DNA isolation was described above. Amplifications were standardized for mitochondrial copy number by simultaneously amplifying a short mitochondrial fragment. We used the following primers for the long mitochondrial DNA product: 5′-CCC AGC TAC TAC CAT CAT TCA AGT AG-3′ for long forward, 5′-GAG AGA TTT TAT GGG TGT AAT GCG GTG-3′ for long backward. For the short mitochondrial DNA product, we used the following primers: 5′-GCA AAT CCA TAT TCA TCC TTC TCA AC-3′ for short forward, GAG AGA TTT TAT GGG TGT AAT GCG GTG-3′ for short backward. The lesion frequency per amplicon was achieved supposing a random distribution of lesions calculated as: Lesion rate = 1–2^∧^(ΔCT Long – ΔCT short) × (1,000 bps/80 bps) (27).

### Transmission Electron Microscopy (TEM)

For TEM analysis, liver tissue was removed and fixed in 2.5% glutaraldehyde in 0.05 M sodium cacodylate buffer (pH 7.4). Portions of glutaraldehyde-fixed liver were further diced into small pieces, post-fixed in 1% osmium tetroxide, and embedded in Epon 812 as described previously (19, 28). Thin sections from selected tissue blocks were cut with an LKB ultramicrotome, stained with uranyl acetate and lead citrate, and examined with a Hitachi electron microscope (Hitachi, Indianapolis, IN). Special emphasis was given to key structural changes associated mitochondrial morphology (29).

### Statistical Analyses

The results were presented as means ± the standard error of the mean (SEM) of at least 5 animals per group. Differences between means of were compared by a 1-way ANOVA followed by the Newman-Keuls multiple comparison test. Differences were considered significant at *P* < 0.05.

## Results

### E-Cigarette (2.4% Nicotine) Exposure Produces DNA Damage Associated With Decreased Hepatic NAD+

**Figure 1:**
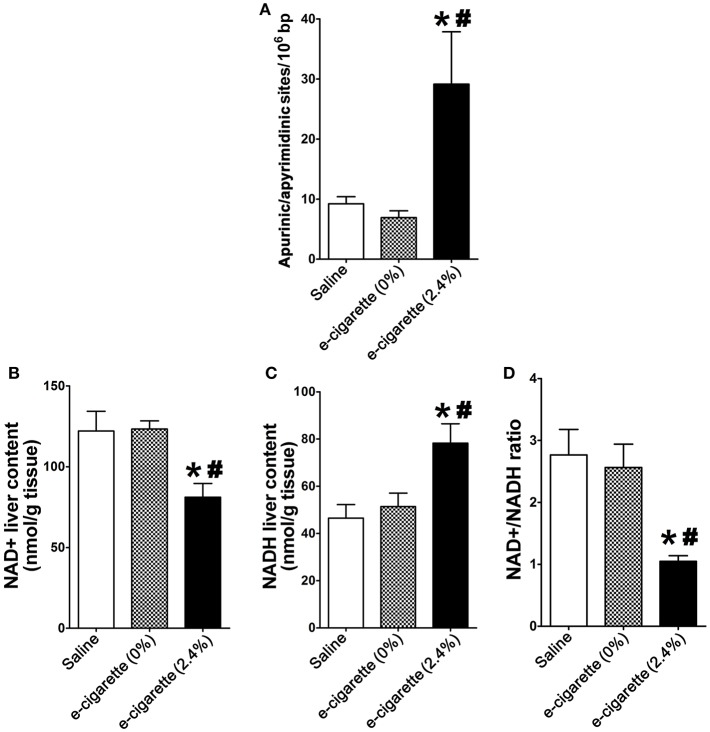
E-cigarette-induced DNA damage is associated with decreased levels of hepatic NAD+. **(A)** E-cigarette (2.4% nicotine), but not E-cigarette (0%) exposure increases AP sites. **(B)** E-cigarette (2.4% nicotine), but not E-cigarette (0%) decreases NAD+ levels. **(C)** E-cigarette (2.4% nicotine), but not E-cigarette (0%) increases NADH levels. **(D)** E-cigarette (2.4% nicotine), but not E-cigarette (0%) decreases NAD+/NADH ratio. *N* = 5 per group, ^*^P < 0.05, saline aerosol vs. e-cigarette (2.4% nicotine). ^#^*P* < 0.05, E-cigarette (0%) vs. e-cigarette (2.4% nicotine).

Figure 1A shows the profiles of AP site induction, respectively, in mice exposed to saline, e-cigarette (0% nicotine), and e-cigarette (2.4% nicotine) for 12 weeks. AP site lesions were increased in mice exposed to e-cigarette (2.4% nicotine). In contrast, in e-cigarette (0%)-exposed animals, the levels of AP site lesions were not significantly changed compared with saline controls. The data suggest that the effects of e-cigarettes on hepatic DNA base damage is due to the presence of nicotine.

Figure 1B shows that mice exposed to e-cigarette (2.4% nicotine) had decreased levels of NAD+ in comparison with both saline-treated (*P* < 0.05) and e-cigarette (0%)-treated mice (*P* < 0.05). NAD+ levels of mice exposed to saline and e-cigarette (0%) were similar. Figure 1C shows that mice exposed to e-cigarette (2.4% nicotine) had increased levels of NADH in comparison with both saline-treated (*P* < 0.05) and e-cigarette (0%)-treated mice (*P* < 0.05). NADH levels of mice exposed to saline and e-cigarette (0%) were similar. The NAD+/NADH ratio of the e-cigarette (2.4% nicotine)-treated ApoE^−/−^ mice was decreased in comparison with saline (*P* < 0.01), without showing any significant changes between saline and e-cigarette (0%) exposed mice (Figure 1D). These results suggest that nicotine is necessary for the effects of e-cigarette (2.4% nicotine) on perturbation of NAD+/NADH ratio.

### E-Cigarette (2.4% Nicotine) Increases Levels of Oxidative Stress

**Figure 2:**
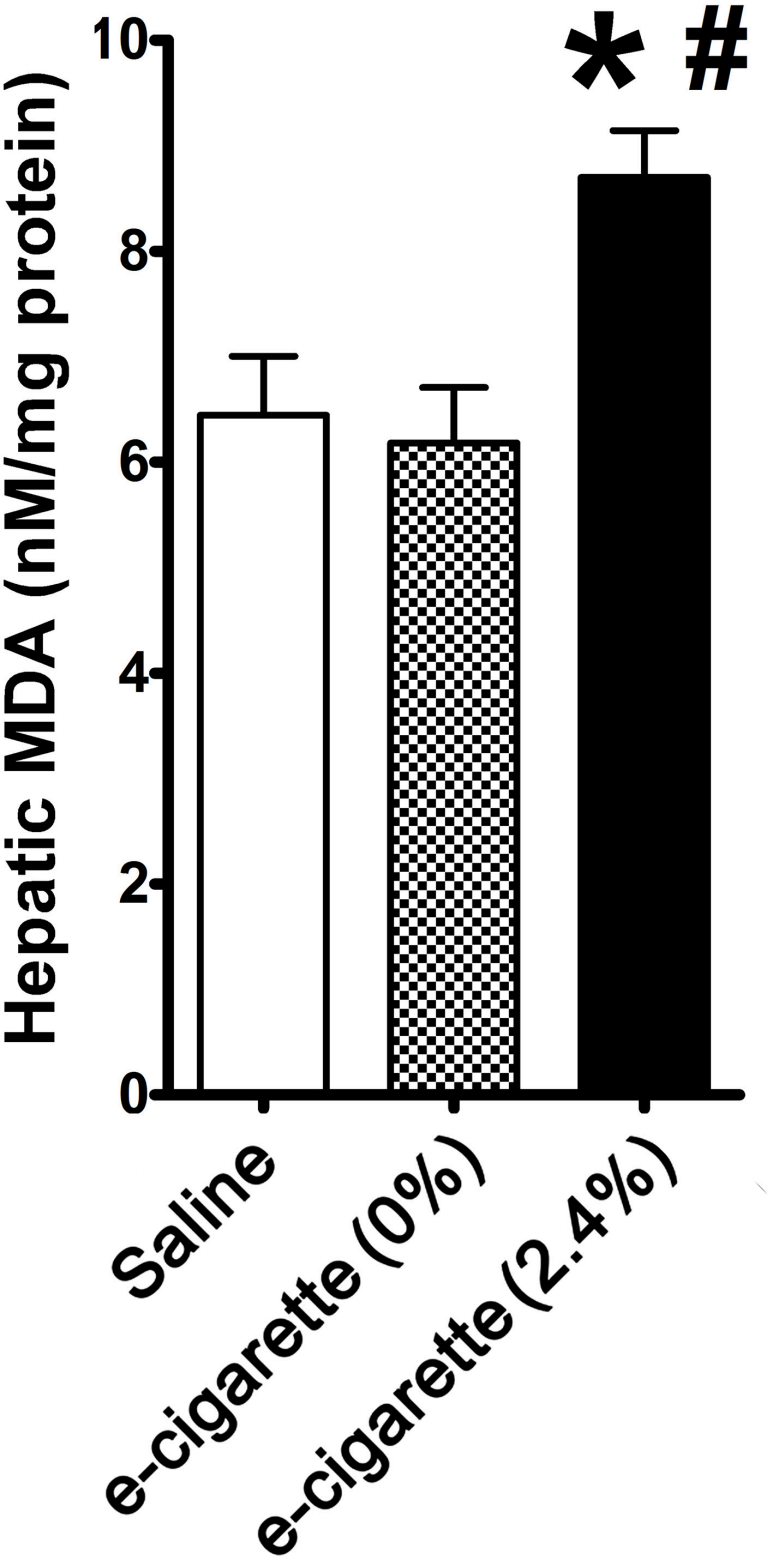
Effects of e-cigarette exposure on oxidative stress in hepatic cells. E-cigarette (2.4% nicotine), but not e-cigarette (0%) increases MDA concentration. *N* = 5 per group, ^*^P < 0.05, saline aerosol vs. e-cigarette (2.4% nicotine). ^#^*P* < 0.05, E-cigarette (0%) vs e-cigarette (2.4% nicotine).

Figure 2 shows that hepatic MDA was increased by 35% in mice treated with e-cigarette (2.4% nicotine) in comparison with both saline-treated (*P* < 0.05) and e-cigarette (0%)-treated mice (*P* < 0.05). Our results show that increased levels of AP site lesions are associated with increased oxidative stress in mice treated with e-cigarette (2.4% nicotine), but not e-cigarette (0%).

### E-Cigarette (2.4% Nicotine) Modulates the PARP1/SIRT1 Axis

PARP-1 activation and consequent NAD+ decrease are a well-established signaling pathway that mediates hepatic cell death and metabolic changes associated with DNA damage (30). After extensive DNA damage, PARP1 has two contrasting roles: (1) PARP1 over-stimulation leads to poly(ADP-ribose) (PAR) synthesis and (2) activation of downstream caspases cause PARP-1 cleavage and inactivation (31). Based on the observations that only e-cigarette containing 2.4% nicotine treatment is associated with increased accumulation of oxidative DNA lesions and decreased hepatic NAD+/NADH, we hypothesized that DNA damage-induced cellular signaling would be increased in mice exposed to e-cigarette. Since we observed increase in AP sites and decease of NAD+/NADH ratio only in e-cigarette -treated mice in the presence, but not in the absence of nicotine, we focused our cellular signaling analysis only on saline- and e-cigarette (2.4% nicotine)-exposed groups.

**Figure 3:**
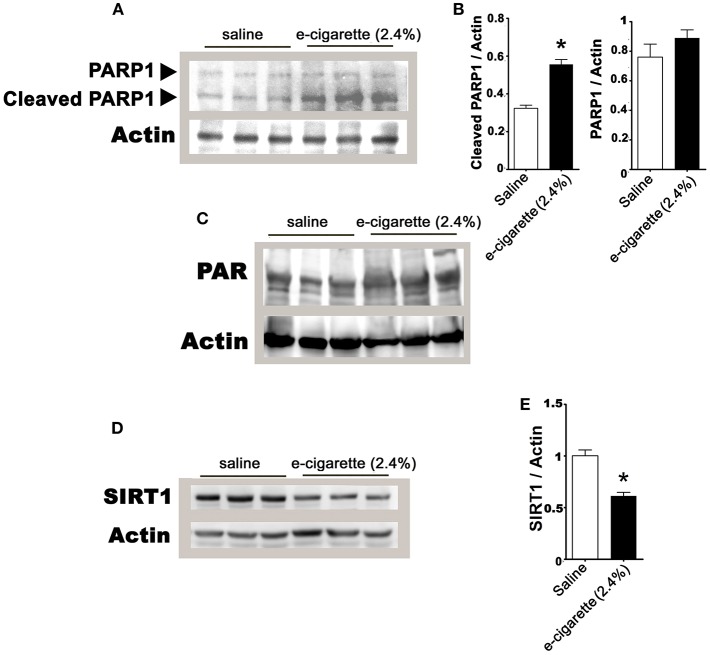
E-cigarette (2.4% nicotine) exposure induces PARP-1 activation. **(A)** Western blot analysis of PARP1. **(B)** Quantification of PARP-1 western blot. **(C)** E-cigarette (2.4% nicotine) increases PAR levels. **(D)** Western blot analysis of SIRT1. **(E)** Quantification of SIRT1 western blot. *N* = 5 per group, ^*^P < 0.05, saline aerosol vs. e-cigarette (2.4% nicotine).

In prior studies using e-cigarettes, we observed hepatic steatosis (21), increased levels of serum FFA (32), DNA damage and oxidative stress only in e-cigarette (2.4% nicotine)-treated mice but not in e-cigarette (0%)-treated mice. In Figures 1, 2, we also found that e-cigarette (0%)-treated mice were similar to saline-treated mice. Therefore, we focused our study of the PARP1/SIRT1 axis only on saline and e-cigarette (2.4% nicotine)-exposed groups. Figure 3A shows that an increase in PARP1 cleavage protein levels in e-cigarette -treated livers increase in comparison with saline-treated control. The quantification of this western blot shows an increase in 75% of the 89 kDa fragment with no and significant changes in the full-length protein, 116 kDa (Figure 3B). We also studied the PARylation levels in liver extracts from mice exposed to e-cigarette. Figure 3C shows increased levels of PARylation in proteins extracts from livers of mice exposed to e-cigarette (250–100 kDa). These results suggest that e-cigarette (2.4% nicotine)-induced DNA damage decreases NAD+ levels possibly via an activation of PARP-1.

PARPs and SIRT1 have an intensive crosstalk not only by competition by NAD+ levels but also by cross-modification and transcriptional regulation (33).Thus, we analyzed the effects of e-cigarette (2.4% nicotine) on SIRT1 protein levels. Figure 3D shows that e-cigarette induced a 28% reduction (*P* < 0.05) in the SIRT1 proteins levels (Figure 3E). Our data suggest, in mice treated with e-cigarettes, the activity of SIRT1 is not only reduced by the depletion of NAD+ levels by PARP1 activity, but also by the reduction of SIRT1 protein levels.

### E-Cigarette (2.4% Nicotine) Induces Hepatic Mitochondrial Dysfunction Markers

**Figure 4:**
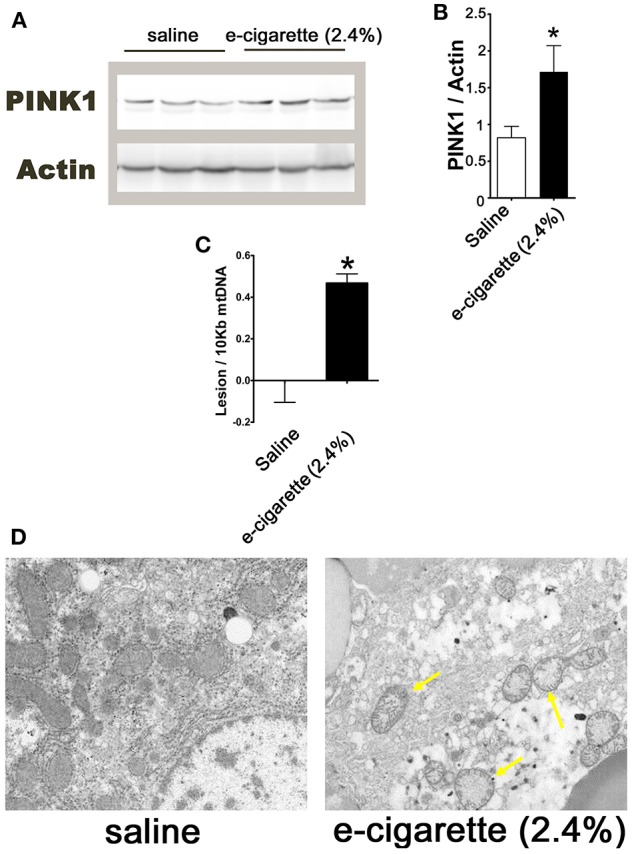
E-cigarette (2.4% nicotine) exposure induces hepatic mitochondrial dysfunction. **(A)** Western blot analysis of PINK1. **(B)** Quantification of PINK1 western blot. **(C)** MtDNA lesions in hepatic cells. **(D)** Representative TEM images of hepatocytes from mice exposed to saline compared to e-cigarette (2.4% nicotine). Arrows indicate vacuolated mitochondria. *N* = 5 per group, ^*^saline aerosol vs. e-cigarette (2.4% nicotine).

Increased levels of PARP1 activity releases poly (ADP-ribose) polymer from the nucleus to the cytosol, leading to depolarization of the mitochondrial membrane potential (34). Mitochondrial membrane depolarization produces the stabilization of PINK1. Figure 4A shows an immune blot of PINK1 of hepatic extracts from saline and e-cigarette (2.4% nicotine)-treated mice. The quantification of this western blot shows a 112% increase (*P* < 0.05) in PINK1 protein levels (Figure 4B). Mitochondrial DNA (mtDNA) is highly sensitive to ROS since it lacks histones and is in close vicinity with the respiratory chain machinery. Figure 4C shows an increase in 0.47 lesion/10,000 bases (*P* < 0.01) in mice exposed to e-cigarette (2.4% nicotine) than that of saline-exposed mice (lesions set at 0). TEM revealed increased mitochondrial vacuolization and a reduction in cellular organelles in e-cigarette (2.4% nicotine)-treated livers compared to saline-treated livers (Figure 4D).

## Discussion

**Figure 5:**
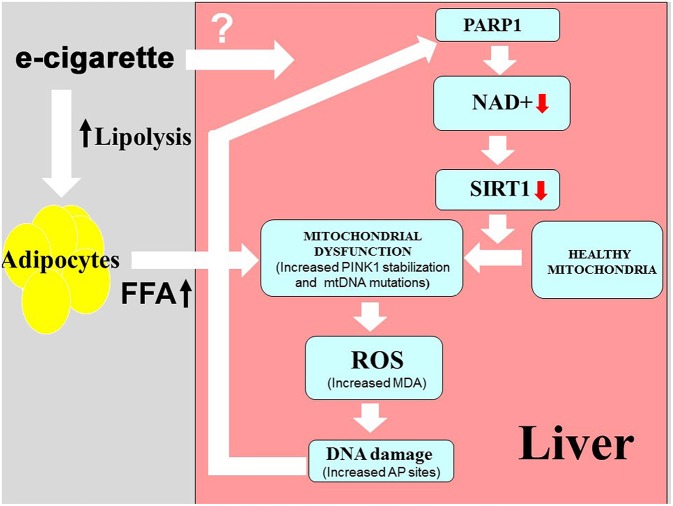
Model for hepatic mitochondrial dysfunction induced by e-cigarette exposure. Area in pink shows mechanisms studied in this paper. The lipolysis shown in the gray area is predicted based on prior studies on nicotine, but has not been directly studied with e-cigarettes.

In order to elucidate the molecular effects of e-cigarettes on the liver, our laboratory developed a mouse model that was exposed to chronic intermittent e-cigarette aerosol in a manner similar to that of human e-cigarette users. Using a mouse model of NAFLD and our newly developed mouse e-cigarette exposure model system, we demonstrated that nicotine in e-cigarettes activates hepatocyte apoptosis and causes hepatic steatosis (21) and increased levels of serum FFA (Espinoza-Derout, 2019) in comparison with the e-cigarette (0%) and saline exposure. The present work provides evidence that: (1) the presence of nicotine in e-cigarettes caused an increase in oxidative DNA damage associated with a depletion of NAD+ levels in the liver of ApoE^−/−^ mice exposed to e-cigarettes; (2) e-cigarettes resulted in PARP1 activation and a decrease in SIRT1 protein levels; and (3) the oxidative stress induced by e-cigarettes produced increased levels of PINK1 and mtDNA mutations, associated with mitochondrial ultrastructural changes. Our model of how e-cigarettes could lead to hepatic mitochondrial dysfunction is given in Figure 5.

PARPs activity is necessary for the ROS induced mitochondrial injury (35). Double KO mice for PARP1 and PARP2 showed mitochondrial enrichment and insulin sensitivity (36). In contrast, increased SIRT1 activity had a protective effect against insulin resistance in the liver in mice exposed to a HFD (37). The inhibition of PARP1 activity improved mitochondrial metabolism through SIRT1 activation, by increasing NAD+ availability (10). In contrast, since SIRT1 has a lower affinity and slower turnover rate for NAD+, decreased activity of SIRT1 did not modulate PARP1 activity (38). However, acetylated PARP1 is active and SIRT1 can bind directly to PARP1 and produce PARP1 inactivation by deacetylation (39). At the transcriptional level, PARP2 can repress the production of SIRT1 (40). In agreement with these concepts, we observed increased levels of oxidative stress is associated with increased PARP1 activity and decreased levels of SIRT1 protein.

The presence of PARylated proteins in liver mitochondria has been described for several groups (41, 42). PARP1 activation leads to decrease in mitochondrial potential (35) and this depolarization may lead to the stabilization of PINK1, thus facilitating its accumulation on the outer mitochondrial membrane, which contribute to the modulation of the clearance of damaged mitochondria (18). In a human hepatocyte cell line, ultrastructural mitochondrial alterations induced by oxidative stress can be ablated by PJ34, a specific inhibitor of PARP1, suggesting that the ultrastructural changes of increased vacuolization of the mitochondria observed in this work are dependent on PARP1 activity. Mitochondrial vacuolization is found following prolonged injury of the cell leading associated with lack of energy production and membrane damage (43). Since mtDNA lacks protection from histones and has closeness to the source of ROS, it is very vulnerable to the oxidative alterations in its coding region (44). Smokers show increased levels of mitochondrial DNA damage in bronchoalveolar lavage tissue (45). Also, animal models of smoking have shown mtDNA damage in the liver (46). Consistently, in mice exposed to e-cigarette (2.4% nicotine), we found increased levels of PINK1, mtDNA and vacuolization of mitochondria.

Although, e-cigarette-induced DNA damage linked to an increase of nitrosamine-associated adducts have been reported in tissues other than the liver (8), the effects of e-cigarettes on oxidative DNA damage in the liver have not been studied. FFA-induced ROS have been postulated as a major mechanism of DNA damage and mitochondrial dysfunction in the liver (47–49). Nicotine induces lipolysis in adipocyte tissue by directly activating nicotinic acetylcholine receptors (50). The activation of acetylcholine receptor subunit α7 by nicotine produce the AMPK activation that leads to release of free fatty acids (FFAs) into the circulation (51). Hepatic steatosis can be produced mostly by FFA delivery from lipolysis of adipose tissue and increased *de novo* lipogenesis (52). We and others have shown that FFA release from adipocytes plays an important role in nicotine plus HFD-induced hepatic steatosis that can be prevented by acipimox, an inhibitor of lipolysis (19, 51, 53). These results, together with our recent findings that e-cigarettes (2.4% nicotine) caused an increase in levels of serum FFA (Espinoza-Derout et al., submitted), most likely suggest a similar mechanism may also be responsible for e-cigarette-induced liver damage. Of note, we did not observe hepatic steatosis or increased serum FFA levels in mice treated with e-cigarettes in the absence of nicotine [(21), Espinoza-Derout et al., submitted], suggesting that nicotine is necessary for e-cigarette (2.4% nicotine)-induce hepatic damage. We propose that the increased oxidative stress observed by e-cigarette (2.4% nicotine) could be due to the increased levels of serum FFA (Figure 5). However, with the data presented in this work, we cannot exclude a direct effect of nicotine or its metabolites in the liver. For instance, nicotine-derived nitrosamines may directly modulate the hepatic metabolism producing steatosis (54).

A potential limitation of our study is that we did not characterize the e-cigarettes effects on PARP2 activity. Although, PARP1 is the best characterize PARP protein and it accounts for approximately 90% of family protein activity (9), PARP2 has been proposed to be a suppressor of SIRT1 transcription by direct regulation of SIRT1 promoter (40). Therefore, a further study of hepatic PARP2 binding to SIRT1 promoter may help answer this question.

In summary, we propose that oxidative stress induced DNA damage produced by e-cigarette containing nicotine exposure is associated with the depletion of NAD+ levels, PARP1 activation, a decrease in SIRT1 activity, together with mtDNA mutations, mitochondrial vacuolization and possible mitochondrial dysfunction. Overall, these results reveal novel insights into the key signaling pathways leading to e-cigarette-induced DNA damage and mitochondrial dysfunction.

## Data Availability

The raw data supporting the conclusions of this manuscript will be made available by the authors, without undue reservation, to any qualified researcher.

## Ethics Statement

This study was carried out in accordance with the recommendations in the Guide for the Care and Use of Laboratory Animals of the National Institute of Health. The Institutional Animal Care and Use Committee on the Ethics of Animal Experiments of Charles R. Drew University of Medicine and Science approved the protocol.

## Author Contributions

JE-D, EB, and KH performed the experiments. JE-D, TF, XS, and AS-H conceived and planned the experiments. JE-D, TF, AS-H, and XS contributed to the interpretation of the results. JE-D, TF, XS, AS-H, YL, and NM wrote the manuscript. TF obtained funding for the project and supervised the project.

### Conflict of Interest Statement

The authors declare that the research was conducted in the absence of any commercial or financial relationships that could be construed as a potential conflict of interest.
